# Intratumoral microbiota omics analysis in head and neck squamous cell carcinoma

**DOI:** 10.3389/fmicb.2025.1711139

**Published:** 2025-11-27

**Authors:** Cuiliu Xie, Qifang Yan, Pan Zhang, Li Zhang, Guowei Yan

**Affiliations:** 1 The Affiliated Stomatological Hospital, Southwest Medical University, Luzhou, China; 2 Luzhou Key Laboratory of Oral and Maxillofacial Reconstruction and Regeneration, Luzhou, China; 3 Department of Stomatology, Traditional Chinese Medicine Hospital of Tongliang District, Tongliang, China

**Keywords:** head and neck squamous cell carcinoma, intratumoral microbiota, 5R 16S rDNA gene sequencing, dysbiosis, metabolism

## Abstract

**Objective:**

The intratumoral microbiota plays a critical role in the progression of head and neck squamous cell carcinoma (HNSCC). This study aimed to comprehensively characterize the intratumoral microbiota in HNSCC and investigate its potential associations with tumor progression and host metabolic functions using a 4-nitroquinoline 1-oxide (4NQO)-induced murine model.

**Methods:**

A HNSCC mouse model was established through administration of 4NQO in drinking water. Histopathological and IHC analyses were performed to confirm tumor development and proliferative activity. The microbial composition of tumor and normal tissues was assessed using 5R 16S rDNA gene sequencing.

**Results:**

Successful induction of HNSCC was confirmed by histopathology and elevated PCNA expression. Significant decreases in microbial α-diversity and altered β-diversity were observed in tumor tissues. Enrichment of genera such as *Aggregatibacter* and *Pseudomonas*, and depletion of *Lactobacillus* and *Acinetobacter*, were characteristic of the intratumoral microbiota. RDA and linear regression revealed a significant correlation between the intratumoral microbiota and PCNA expression. Predictive functional analysis indicated alterations in metabolic pathways, including fatty acid biosynthesis and nucleotide metabolism, in the tumor microenvironment.

**Conclusion:**

These findings demonstrate that intratumoral microbiota dysbiosis is closely associated with HNSCC progression. The study establishes a foundational murine model for further mechanistic research and suggests the potential of the intratumoral microbiota as a biomarker or therapeutic target in HNSCC.

## 1 Introduction

Head and neck squamous cell carcinoma (HNSCC) is the seventh most common malignancy worldwide, with an annual incidence of 890,000 cases and a five-year survival rate that remains below 50% (Chow, 2020). This heterogeneous group of cancers arises from the mucosal epithelium of the oral cavity, pharynx, and larynx. Their development is strongly linked to several major risk factors, most notably tobacco, alcohol, and infection with high-risk types of human papillomavirus (Solomon et al., 2018). Despite advances in multimodal therapy, clinical outcomes and high recurrence rates for HNSCC remain heterogeneous. Therefore, novel diagnostic markers and new therapeutic strategies for HNSCC are urgently needed to improve the prognosis and treatment response of this population.

Evidences indicate that the microbiome is associated with the pathogenesis and progression of various malignancies, including HNSCC (Frank et al., 2022; Fu et al., 2022). The salivary microbiota has been linked to HNSCC (Lou et al., 2025), suggesting its potential role as a biomarker for HNSCC diagnosis and treatment. However, most current studies on HNSCC have focused primarily on salivary samples rather than on microbial communities directly derived from tumor tissues. Therefore, investigating the microbiota obtained directly from tumor tissues may provide critical insights into microbiome–host interactions and their functional roles in HNSCC tumorigenesis.

In this study, we established a spontaneous HNSCC mouse model using 4-nitroquinoline-1-oxide (4NQO) induction, followed by comprehensive profiling of the intratumoral microbial characteristics using 5-region (5R) 16S rDNA gene sequencing.

## 2 Materials and methods

### 2.1 4NQO-induced HNSCC mouse model

Female 12-week-old C57BL/6 mice from the Experimental Animal Center of Southwest Medical University were administered 4NQO (Sigma, United States) in the drinking water at a concentration of 100 μg/mL. Fresh drinking water containing 4NQO was supplied weekly. Following 14 weeks of 4NQO exposure to induce HNSCC, the treatment was discontinued and mice were maintained on regular drinking water (Lou et al., 2025). Age-matched control mice received normal drinking water without 4NQO throughout the experiment. After being anesthetized with an intraperitoneal injection of tribromoethanol (400 mg/kg), the mice were euthanized via cervical dislocation. Tumor and normal tissue specimens were aseptically collected, rinsed with sterile phosphate-buffered saline (PBS) and iodine solution, after which the outer layer (approximately 2–3 mm) of the tissue was surgically removed. This procedure ensured that only the inner portion of the tumor was used for subsequent DNA extraction and analysis. The experimental animals were supplied by the Experimental Animal Center of the Department of Basic Medicine, Southwest Medical University. Ethical oversight and approval for the animal studies were granted by the Affiliated Stomatological Hospital, Southwest Medical University Ethical Committee (No. 20191227001). Animal studies were performed in adherence to guidelines of the Care and Use of Laboratory Animals.

### 2.2 Histological analysis

Tongue tissues were fixed with 4% paraformaldehyde and embedded in paraffin, sectioned and stained with hematoxylin and eosin (H&E) (Beyotime, Shanghai, China) according to the manufacturer’s instructions. The sections were scanned and observed using pathology slide scanner (KF-PRO-002, Germany).

### 2.3 Immunohistochemical analysis

Tongue tissue specimens were immobilized in 4% paraformaldehyde, embedded in paraffin, and sectioned for immunohistochemical (IHC) analysis. IHC was carried out with a Rabbit Enhanced Polymer Detection System (ZSGB-BIO, PV-9001, Beijing, China). Sections were incubated with a primary antibody targeting Proliferating cell nuclear antigen (PCNA) (13110S, Cell Signaling Technology, Danvers, Massachusetts, United States) at a dilution of 1:4000. Antigen–antibody binding was detected using DAB chromogen (ZSGB-BIO, ZLI-9018, Beijing, China). Finally, all stained sections were digitized using a pathology slide scanner (KF-PRO-002, Germany) for subsequent evaluation.

### 2.4 DNA extraction and 5R 16S rDNA gene sequencing

Total microbial community DNA was extracted from tumor and normal tongue tissue samples as previously described (Yan et al., 2025). The quality of the extracted DNA was assessed by 1% agarose gel electrophoresis, while concentration and purity were determined using a NanoDrop 2000 spectrophotometer. To minimize interference from host DNA and enhance the detection of microbiota in samples characterized by low microbial biomass and high host-derived nucleic acids, 5R 16S rDNA gene sequencing (Majorbio Bio-pharm Technology Co. Ltd., Shanghai, China) was used in this study. Specific primers with barcodes were synthesized targeting five variable regions of the 16S rDNA gene (V2, V3, V5, V6, V8) according to the designated sequencing areas. The primer sequences for each of the five regions (5R) are shown in Table 1. Multiplex polymerase chain reaction (PCR) amplification was subsequently performed. The negative controls (DNA extraction controls and no- template PCR amplification controls) were also included. Following amplification, the PCR products were quantified using the Quantus™ Fluorometer. Subsequently, amplicon pools were readied for sequencing, with the size and quantity of the amplicon library evaluated on an Agilent 2100 Bioanalyzer (Agilent Technologies, United States) and using the NEXTFLEX Rapid DNA-Seq Kit for Illumina 16S Metagenomic Sequencing Library (Kapa Biosciences, Woburn, MA, United States), respectively. Paired-end sequencing is performed using the Illumina NovaSeq 6000 platform.

**Table 1:** Primer sequences used for 5R 16S rDNA gene sequencing.

Sequencing areas	Forward primer	Reverse primer
V2	TGGCGAACGGGTGAGTAA	CCGTGTCTCAGTCCCARTG
V3	ACTCCTACGGGAGGCAGC	GTATTACCGCGGCTGCTG
V5	GTGTAGCGGTGRAATGCG	CCCGTCAATTCMTTTGAGTT
V6	GGAGCATGTGGWTTAATTCGA	CGTTGCGGGACTTAACCC
V8	GGAGGAAGGTGGGGATGAC	AAGGCCCGGGAACGTATT

### 2.5 Bioinformatics analysis

The sequencing data was analyzed using the Short Multiple Regions Framework (SMURF) pipeline with the Greengenes database (2013 version) (Nejman et al., 2020). To reduce noise variation at very low abundance levels, samples with fewer than 1,000 normalized reads (including negative controls) and species with relative abundances below 10^−4^ were excluded from further analysis. To address the most common background contaminants, any species detected in over 30% of either the negative DNA/PCR/Sequencing controls were completely excluded from the dataset. Amplicon sequence variants (ASVs) were rigorously refined to characterize microbial community composition. Alpha diversity (α-diversity, including the Sobs, Chao, Shannon, and Simpson indices) and beta diversity (β-diversity) were evaluated from species-level abundance profiles. Rarefaction curves based on the Sobs and Shannon indices were constructed to evaluate sampling sufficiency. Principal component analysis (PCA) and principal co-ordinates analysis (PCoA) based on Bray-Curtis distance was carried out to visualize the beta diversity between different groups. Community composition analysis was visualized using Python (version 2.7). Linear discriminant analysis effect size (LEfSe) is used to identify differentially abundant taxa across multiple phylogenetic levels (phylum, class, order, family, genus, and species), with thresholds set at a linear discriminant analysis (LDA) score > 4 and *p* < 0.05. Statistical comparisons of taxonomic abundances between the two groups at the genus and species levels were performed using Wilcoxon rank-sum tests. Fold change (FC) was calculated as the ratio of the mean abundance of a microbial taxon in the control group to its mean abundance in the tumor group. RDA, linear regression, and MaAsLin2 analysis were employed to determine the associations between microbial compositional features and both HNSCC tumorigenesis and proliferative capacity. The functional metabolic potential was predicted using PICRUSt2, with inferred pathway abundances based on the MetaCyc database and ortholog clusters profiled against the COG database.

### 2.6 Quantitative PCR

Total genomic DNA was extracted from mouse tumor and normal tissues with Genomic DNA Purification Mini Spin Kit (Beyotime, China). The sequences of primers used for quantitative PCR (qPCR) are shown in Table 2. The qPCR was performed by QuantStudio™ 7 Flex Real-Time PCR System (Thermo Fisher Scientific) using SYBR Green^®^ Premix DimerEraser™ (Takara, Japan). Normalization of the target bacterium’s absolute copy number was achieved by dividing it by the total bacterial 16S rRNA gene copy number in the same sample, as quantified via a separate qPCR assay with universal 16S rRNA gene primers.

**Table 2:** The primer sequences used for qPCR.

Gene	Forward 5′-3′	Reverse 5′-3′
*Enterococcus faecalis*	ACGAAAGTCTGACCGAGCAA	CGTCAGGGGACGTTCAGTTA
*Acinetobacter baumannii*	AGAAAGCAGGGGATCTTCGG	ATCCTCTCAGACCCGCTACA
*Enterococcus faecium*	GTTGGTGAGGTAACGGCTCA	TGCTCGGTCAGACTTTCGTC
*Staphylococcus aureus*	ACGTAGGTGGCAAGCGTTAT	CGCATTTCACCGCTACACAT
*Pasteurella pneumotropica*	GGCAGGCTTAACACATGCAA	AAGCATTACTCACCCGTCCG
*Bacillus infernus*	TTGCTTTTGATCGTCAGCGG	TAGCTCACGTTTCCGCAAGT
*16S rRNA*	AGAAAATCTGGCACCACACCT	GATAGCACAGCCTGGATAGCA

### 2.7 Statistical analysis

Data are presented as mean ± standard deviation (SD). Comparisons between groups were performed using a two-tailed Student’s *t*-test. The significance of beta diversity was assessed by ANOSIM. All statistical analyses were carried out with GraphPad Prism version 9.0 (La Jolla, United States) and R software (version 3.5.2). *p* < 0.05 was considered statistically significant, with asterisks indicating the level of significance (*p < 0.05, **p < 0.01, ***p < 0.001).

## 3 Results

### 3.1 Construction of a 4NQO-induced HNSCC model

**Figure 1:**
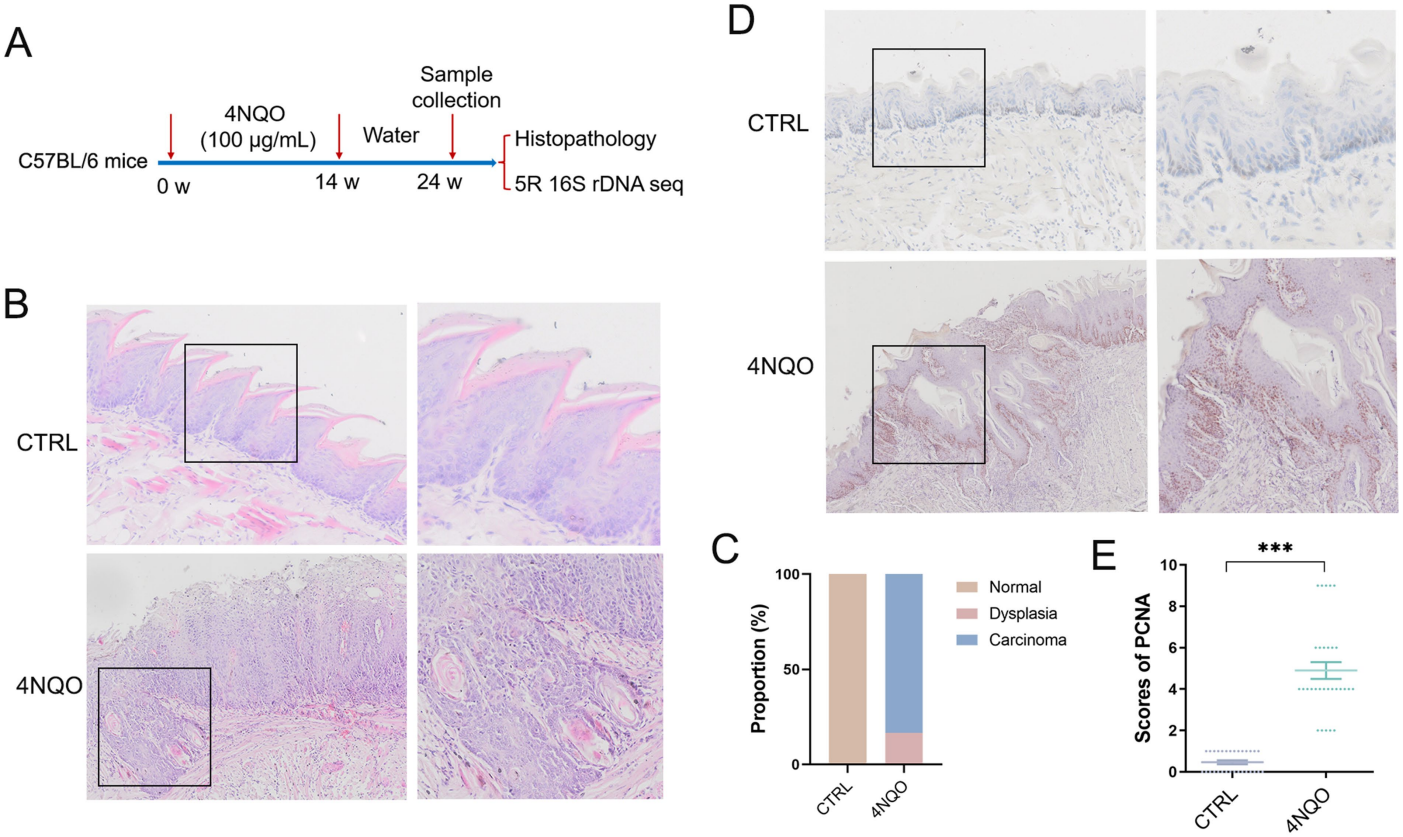
Construction of a 4NQO-induced HNSCC model. **(A)** Schematic overview of the 4NQO-induced HNSCC model (4NQO group, *n* = 6; CTRL group, *n* = 6). **(B)** Representative images of H&E staining in mouse tongue tissue. **(C)** Prevalence of normal, dysplasia and carcinoma in the tongue tissue. **(D)** Representative images of immunohistochemcal staining of PCNA positive cells in the tongue tissue. **(E)** The score for the number of PCNA positive cells in the tongue tissue (*n* = 5 fields from 6mice). Data are expressed as mean ± SD. Statistical significance was determined by two-tailed Student’s *t*-test. 4NQO, 4-nitroquinoline-1-oxide; PCNA, proliferating cell nuclear antigen. ***p < 0.001.

4NQO was used to induced HNSCC model. 12-week-old mice were administered for 14 weeks with 4NQO in the drinking water, followed by a 10-week period of regular water consumption. All animals were euthanized at 24 weeks for tissue collection and subsequent analysis (Figure 1A). Histological staining of tongue tissue showed that the presence of squamous cell carcinoma and dysplastic epithelia in 4NQO group (Figures 1B,C). IHC staining revealed that 4NQO treatment induced a marked upregulation of PCNA expression (Figures 1D,E). These results demonstrate the successful establishment of a 4NQO-induced mouse model of HNSCC.

### 3.2 Decreased intratumoral microbiota diversity in HNSCC

**Figure 2:**
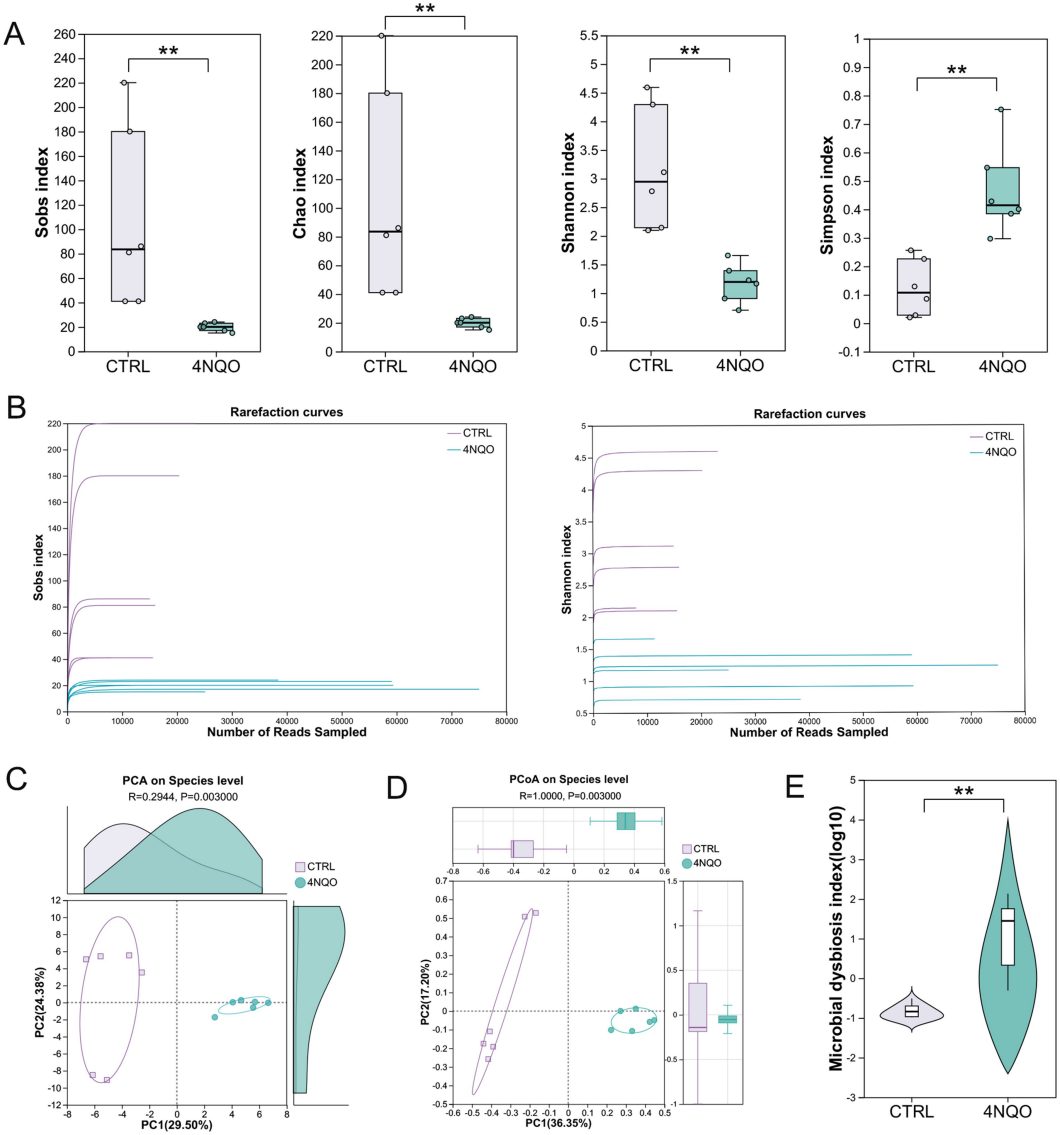
Decreased intratumoral microbial diversity in HNSCC. **(A)** The alpha-diversity indices (including Sobs, Chao, Shannon, and Simpson indices) of the 4NQO group and CTRL group (4NQO group, *n* = 6; CTRL group, *n* = 6). The significance of alpha diversity was assessed by a two-tailed Wilcoxon rank-sum test. **(B)** Rarefaction curves were used to evaluate whether the sequencing depth per sample was sufficient. **(C)** PCA analysis. The significance of beta diversity was assessed by ANOSIM. **(D)** PCoA analysis. The significance of beta diversity was assessed by ANOSIM. **(E)** Analysis of differences in MDI. A two-tailed Wilcoxon rank-sum test was used. The Benjamini-Hochberg FDR was applied for multiple testing correction. 4NQO, 4-nitroquinoline-1-oxide; PCA, principal component analysis; PCoA, principal coordinates analysis; MDI, microbial dysbiosis index. **p < 0.01.

16S rRNA gene sequencing was used to characterize the microbial composition of tumor and normal tissue samples from mice in the 4NQO-treated and control (CTRL) groups. Microbial community α-diversity significantly differed between tumor and normal tissues. Specifically, species richness (as indicated by the Sobs and Chao indices) was significantly lower in the 4NQO-treated group than in the control group. Concurrently, species evenness (as indicated by the Simpson index) was higher. This overall reduction in community complexity resulted in a decrease in α-diversity, as reflected by the Shannon index (Figure 2A). The rarefaction curves based on the Sobs and Shannon indices revealed asymptotic trends, indicating an adequate sequencing depth (Figure 2B). Regarding β-diversity, both PCA and PCoA analyses based on Bray-Curtis distance revealed significant differences at the species level between the 4NQO-treated group and the CTRL group (Figures 2C,D). The microbial dysbiosis index (MDI) also confirmed this significant difference (Figure 2E). These results demonstrate that intratumoral microbiota diversity (α-diversity and β-diversity) is significantly altered in HNSCC.

### 3.3 Differences in the composition of characteristic intratumoral microbiota associated with HNSCC

**Figure 3:**
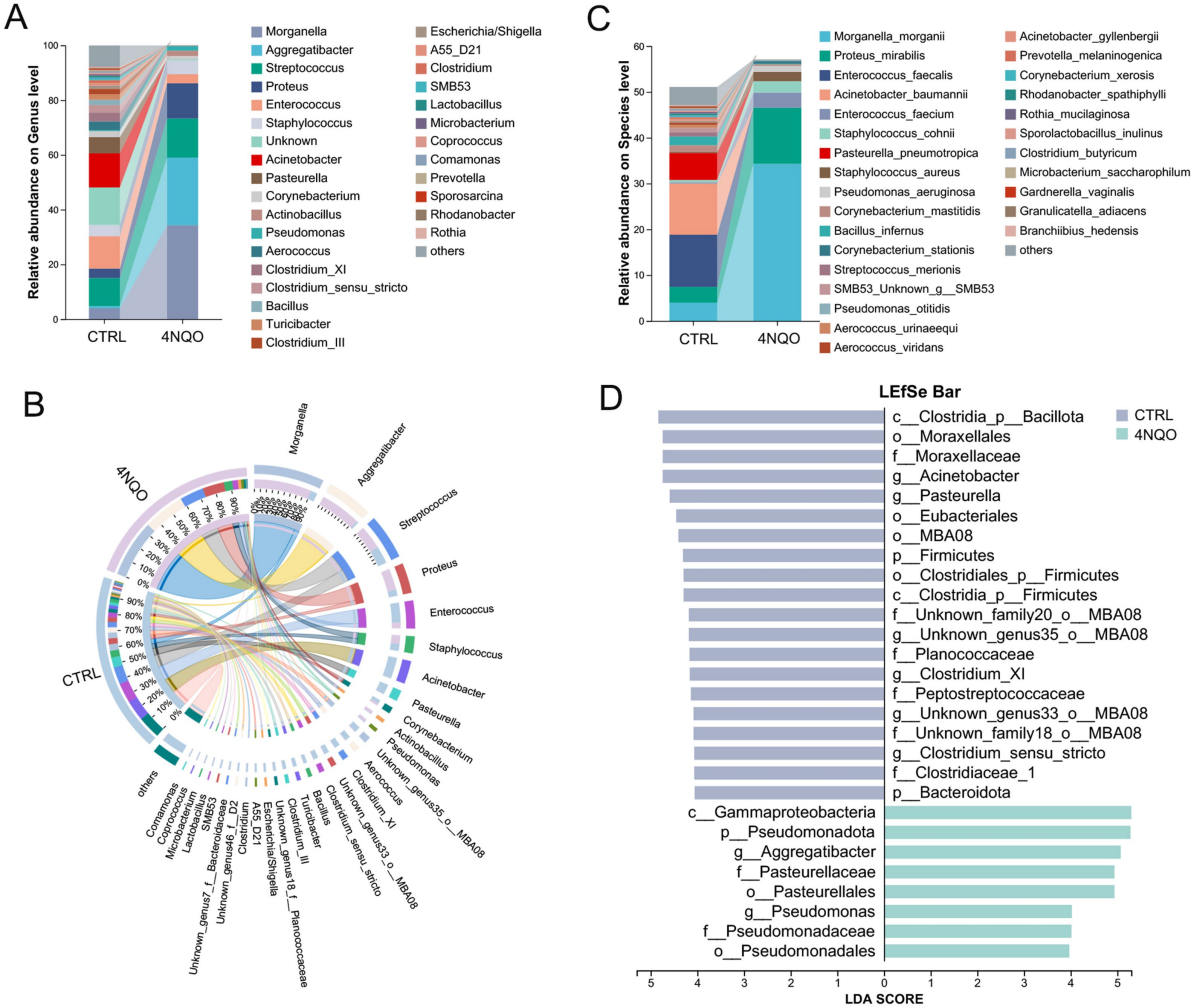
Differences in the composition of characteristic intratumoral microbiota associated with HNSCC. **(A)** The composition of the microbiota at the genus level in the 4NQO group and CTRL group (4NQO group, *n* = 6; CTRL group, *n* = 6). **(B)** A Circos graph showing the distribution of microbial species at the genus level in the 4NQO group and CTRL group. **(C)** The composition of the microbiota at the species level in the 4NQO group and CTRL group. **(D)** LEfSe Bar chart identified the specific microbes with the top abundance that characterized each group (LDA score > 4 and *p* < 0.05). 4NQO, 4-nitroquinoline-1-oxide; LEfSe, linear discriminant analysis effect size; LDA, linear discriminant analysis.

Next, we analyzed the differences of intratumoral microbiota composition. Pronounced differences were observed in the genus-level abundance of the microbiota between tumor and normal tissues. Specifically, in the 4NQO-treated group, the relative proportion of *Morganella*, *Aggregatibacter* and *Proteus* increased, while *Enterococcus*, *Acinetobacter*, and *Pasteurella* decreased (Figure 3A). The Circos plot further confirmed the associations between samples and microbial species, and effectively illustrated the dominance of specific taxa within each group (Figure 3B). At the species level, *Morganella morganii*, *Proteus mirabilis, Enterococcus faecium and Staphylococcus cohnii* showed increased proportion in the 4NQO-treated group, whereas *Acinetobacter baumannii* and *Pasteurella pneumotropica* were less abundant (Figure 3C). Furthermore, LEfSe was used to identify taxonomic differences between microbial communities. The analysis revealed that the 4NQO-treated group was characterized by enriched ASVs belonging to the genera *Aggregatibacter* and *Pseudomonas,* and the *Pasteurellaceae* and *Pseudomonadaceae* families compared with the CTRL group (Figure 3D). These results indicate a significant shift in the community composition of the intratumoral microbiota in HNSCC.

### 3.4 Abundance analysis of characteristic HNSCC-associated intratumoral microbiome

**Figure 4:**
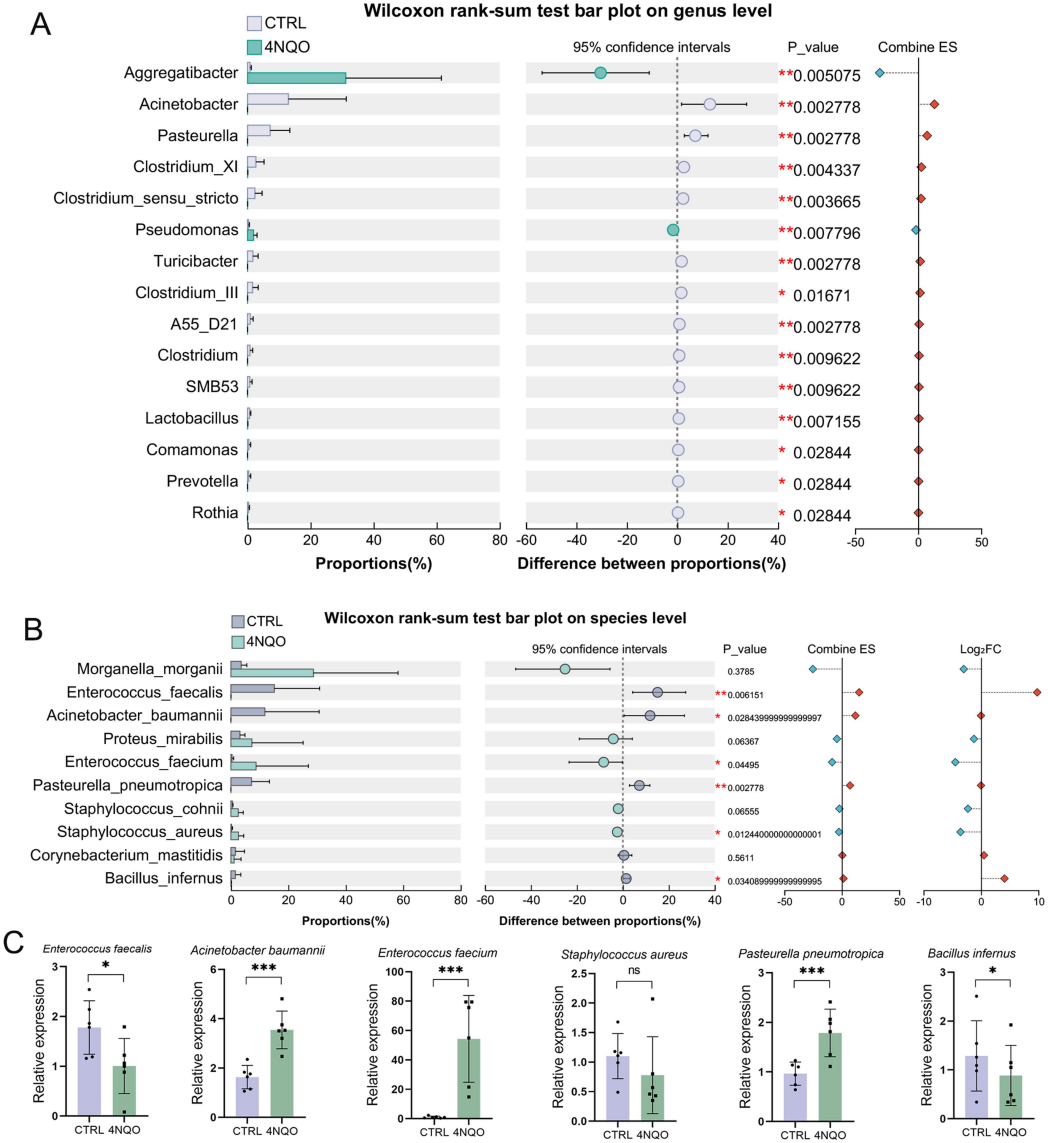
Abundance analysis of characteristic HNSCC-associated intratumoral microbiome. **(A)** Differentially abundant bacterial genera between tumor and normal tissues were identified (4NQO group, *n* = 6; CTRL group, *n* = 6). **(B)** Differentially abundant bacterial between tumor and normal tissues were identified at species level. A two-tailed Wilcoxon rank-sum test was used for hypothesis testing and corrected for multiple comparisons using the Benjamini-Hochberg FDR procedure. Confidence intervals (CIs) were calculated using the bootstrap method (95% CI). **(C)** The significantly altered bacterial species were validated by qPCR. 4NQO, 4-nitroquinoline-1-oxide; FC, fold change. *p < 0.05, **p < 0.01.

We further explored differentially abundant bacteria between the 4NQO-treated and CTRL groups. Bacterial taxa that differed significantly between groups were identified using the Wilcoxon rank-sum test. At the genus level, *Aggregatibacter* and *Pseudomonas* were significantly more abundant in the 4NQO-treated group (*p* < 0.05). In contrast, *Acinetobacter*, *Pasteurella*, *Clostridium*, *Turicibacter*, *Lactobacillus*, *Comamonas*, *Prevotella*, and *Rothia* were less abundant in the 4NQO-treated group (*p* < 0.05) (Figure 4A). At the species level, bacteria such as *Acinetobacter baumannii*, *Pasteurella pneumotropica*, and *Bacillus infernus* were significantly more abundant in the CTRL group, while in the 4NQO-treated group, they were significantly reduced (*p* < 0.05). In contrast, *Enterococcus faecium* and *Staphylococcus aureus* were significantly increased in the 4NQO-treated group (Figure 4B). The significantly altered bacterial species were further validated by qPCR, and the results were consistent with the 5R 16S rRNA sequencing data (Figure 4C). These differences provide important evidence for further investigation into the role of intratumoral microbiota in the development of HNSCC.

### 3.5 Correlation analysis between intratumoral microbiota and proliferative capacity in HNSCC

**Figure 5:**
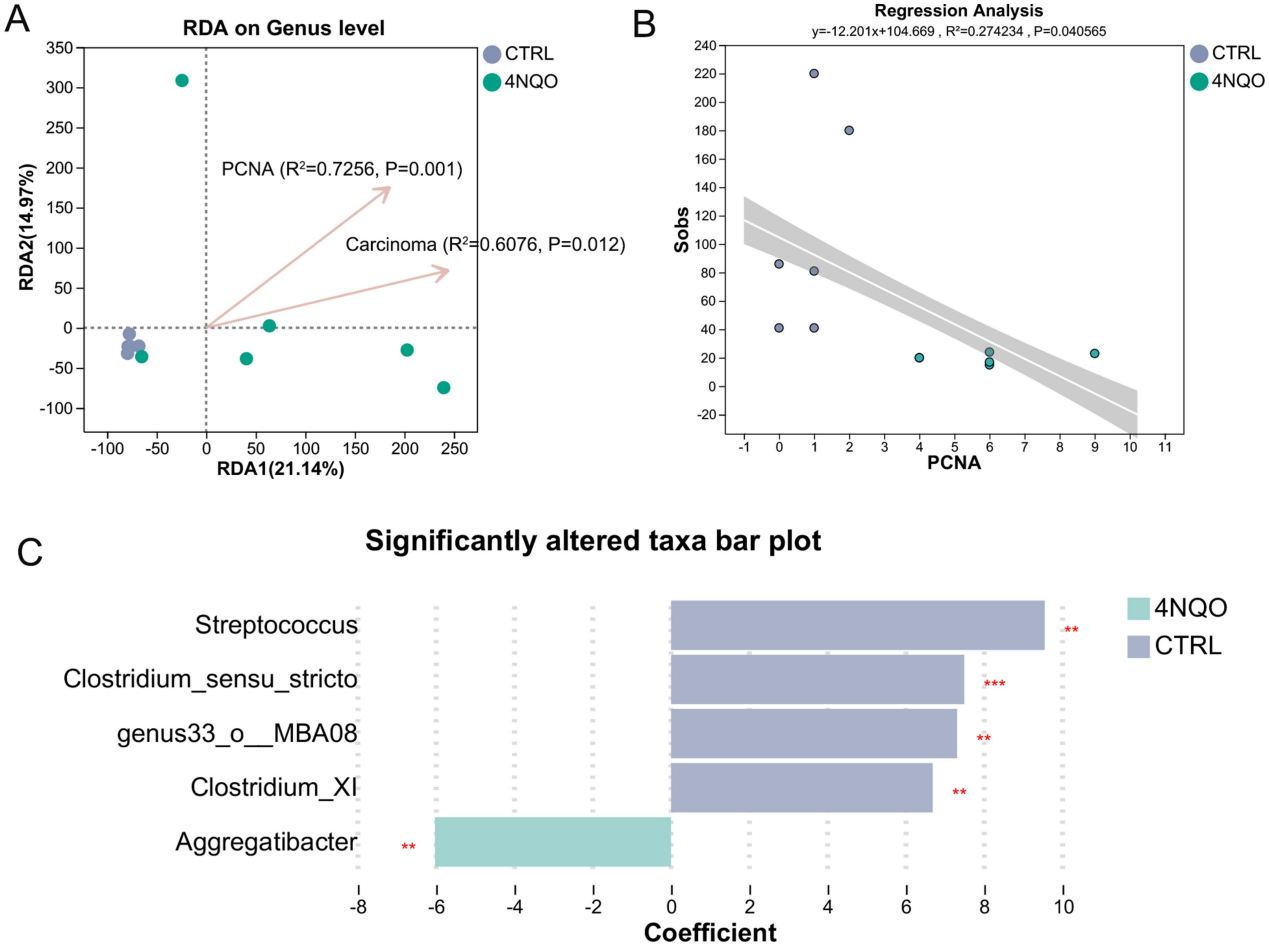
Correlation analysis between intratumoral microbiota and proliferative capacity in HNSCC. **(A)** RDA/CAA analysis showed the degree of influence of clinical factors on microbiota composition, with individual points in the figure indicating sample tissues and arrows indicating clinical factors. **(B)** Linear Regression analysis showed the correlation between environmental factors and the structure of microbial communities. **(C)** MaAsLin2 analysis revealed microbial taxa that were significantly associated with environmental phenotypes. 4NQO, 4-nitroquinoline-1-oxide. **p < 0.01, ***p < 0.001.

We performed RDA analysis to identify associations between the intratumoral microbiota and clinical indicators (including PCNA expression and tumor incidence). The results revealed a significant correlation between clinical indicators (including PCNA expression and tumor incidence) and the intratumoral microbiota in HNSCC (Figure 5A). Linear regression analysis further confirmed a significant correlation between PCNA expression and the structure of the intratumoral microbiota in HNSCC (Figure 5B). Specifically, at the genus level, *Aggregatibacter* was positively correlated with PCNA expression. *Streptococcus* and *Clostridium* showed a strong negative correlation with PCNA expression (Figure 5C). These results demonstrate a significant correlation between the intratumoral microbiota and the progression of HNSCC.

### 3.6 Prediction of functional pathways associated with HNSCC characteristic intratumoral microbiota

**Figure 6:**
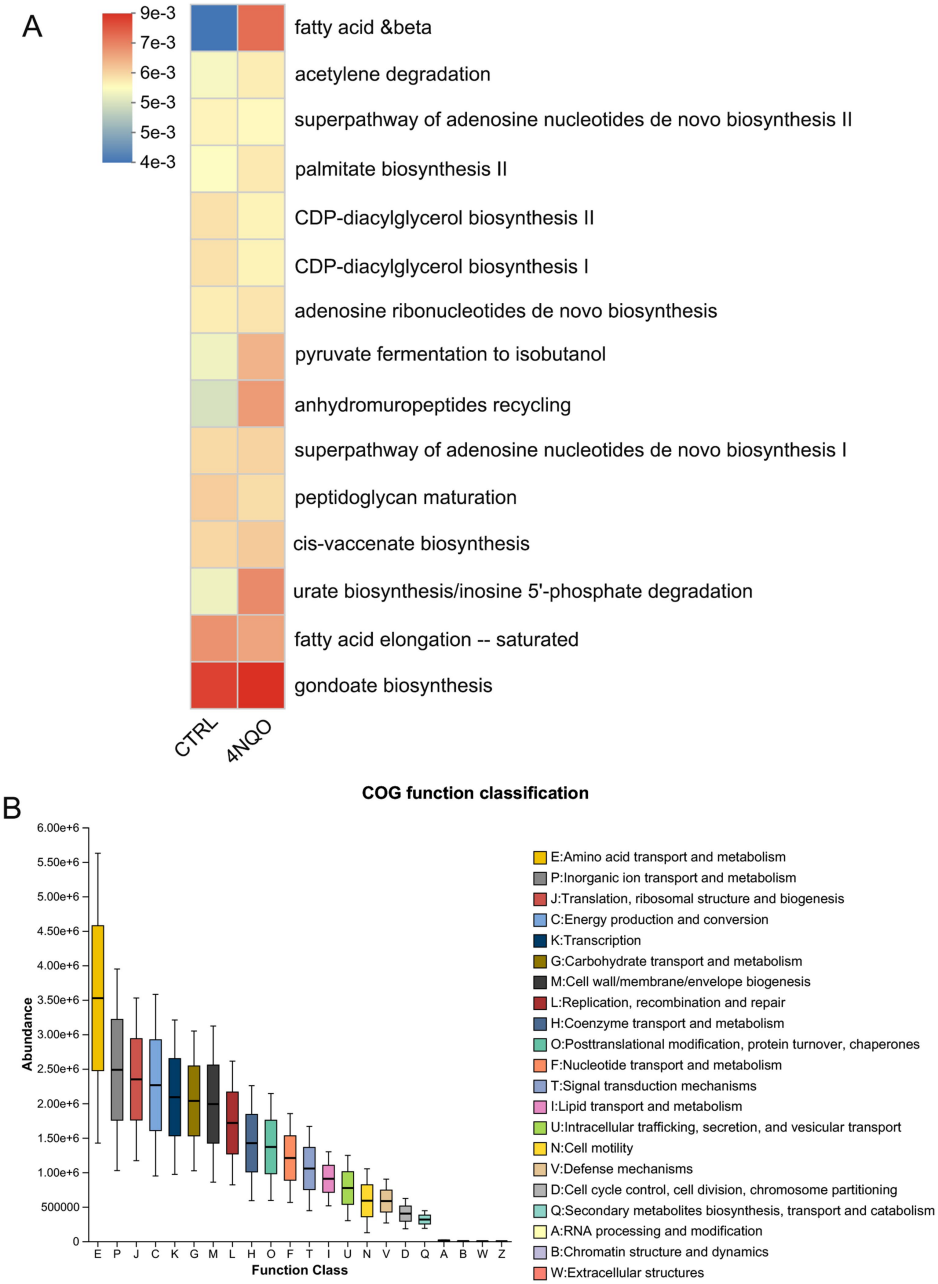
Prediction of functional pathways associated with HNSCC characteristic intratumoral microbiome. **(A)** PICRUSt2 combined with the MetaCyc database to predict the function of bacterial microbiota in tissues. **(B)** PICRUSt2 combined with the COG database to predict the function of bacterial microbiota in tissues. 4NQO, 4-nitroquinoline-1-oxide; MetaCyc, metabolic cyclopedia database; COG, clusters of orthologous genes.

PICRUSt2 analysis was employed to further predict the gene functions of the microbiota. By linking taxonomic data to functional profiles, this approach provides insight into the metabolic capabilities of the microbiome and helps clarify potential mechanisms of microbiota-host interactions. Using gene function annotation from MetaCyc and COG databases, we performed differential analysis based on ANOVA combined with the Tukey–Kramer *post hoc* test to identify the top 15 significantly differential metabolic pathways between the 4NQO-treated group and the CTRL group. MetaCyc analysis revealed differences between the two groups in pathways related to fatty acid β-oxidation, acetylene degradation, adenosine nucleotides *de novo* biosynthesis, palmitate biosynthesis, CDP-diacylglycerol biosynthesis, and adenosine ribonucleotides de novo biosynthesis (Figure 6A). In COG database analysis, we identified significant differences in enzymes related to amino acid transport and metabolism, inorganic ion transport and metabolism, translation, ribosomal structure and biogenesis, energy production and conversion, and carbohydrate transport and metabolism between the two groups (Figure 6B). These results indicate that the intratumoral microbiota is associated with the host’s metabolic systems.

## 4 Discussion

The composition of the intratumoral microbiota has been shown to play a regulatory role in the progression of various malignant tumors, including colorectal cancer, hepatocellular carcinoma, pancreatic cancer, adrenocortical carcinoma, and intrahepatic cholangiocarcinoma (Fu et al., 2022; Li et al., 2023; Liu et al., 2024; Riquelme et al., 2019; Xue et al., 2024). In contrast to other malignancies, studies investigating the intratumoral microbiota in HNSCC are still limited. In this study, we comprehensively characterized the intratumoral microbiota in a 4NQO-induced HNSCC murine model and revealed its significant associations with tumor progression. Our principal findings demonstrate that (1) HNSCC tumorigenesis is associated with a significant decrease in intratumoral microbiota diversity and distinct shifts in microbial composition; (2) specific bacterial genera and species are significantly enriched or depleted in tumors; (3) the altered microbiota is correlated with proliferative activity, as indicated by PCNA expression; and (4) predictive functional profiling suggests that the intratumoral microbiota may influence host metabolic pathways, potentially contributing to tumor microenvironment remodeling and HNSCC pathogenesis.

Currently, research on HNSCC has predominantly focused on the salivary microbiome (Lou et al., 2025; Mäkinen et al., 2023), while studies directly investigating the intratumoral microbiota remain limited. A study utilizing Cancer Microbiome Atlas (TCMA) and The Cancer Genome Atlas (TCGA) database has described the distribution of the intratumoral microbiota in HNSCC (Hamada et al., 2023). There is an urgent need to further explore the role and underlying mechanisms of the intratumoral microbiota in HNSCC using animal models. The observed reduction in microbial α-diversity within tumor tissues is consistent with findings in other solid tumors, such as colorectal and pancreatic cancers (Long et al., 2024; Riquelme et al., 2019). This phenomenon suggests that the tumoral niche selects for a specific microbial community that may favor survival under conditions of hypoxia, inflammation, and altered metabolism (Rodvold et al., 2012). The β-diversity analysis further confirmed that the overall microbial community structure in tumors is fundamentally different from that in normal tissues, underscoring the notion that HNSCC harbors a unique microbiome.

Consistent with previous analyses of the TMA and TCGA databases (Hamada et al., 2023), the abundance of several bacterial genera, including *Acinetobacter*, *Lactobacillus*, and *Rothia*, was significantly altered at the genus level. Notably, we identified several taxa with significant abundance alterations in tumor tissues. Genera such as *Aggregatibacter* and *Pseudomonas* were enriched in the 4NQO-treated group. *Aggregatibacter*, in particular, is a known oral pathobiont associated with periodontal disease and has been implicated in promoting inflammation and epithelial disruption, processes that are central to HNSCC development (Henderson et al., 2010; Salman et al., 2025). Conversely, the depletion of commensals like *Acinetobacter*, *Lactobacillus* and *Clostridium* in tumors may indicate a loss of protective, anti-inflammatory microbes that help maintain mucosal homeostasis (Mattanovich et al., 2021; O'Callaghan and O'Toole, 2013; Daniel and Ridlon, 2025). These compositional changes suggest a shift from a health-associated commensal community to a pro-inflammatory, potentially pro-tumorigenic microbiome. It is notable that tumors are spatially heterogeneous, and that the altered microbial features may be dominated by populations from specific regions. Future studies employing techniques like spatial profiling or micro-dissection will be crucial to unravel the precise spatial architecture of the intratumoral microbiota.

Furthermore, our study revealed a significant correlation between the intratumoral microbiota and proliferative activity, as measured by PCNA expression. The positive correlation between *Aggregatibacter* and PCNA and the negative correlation of *Streptococcus* and *Clostridium* with PCNA suggest that specific microbes may directly or indirectly modulate host cell proliferation. This aligns with emerging evidence that bacteria play a critical role in the initiation and progression of cancer by modulating the immune microenvironment and tumor-related signaling pathways, ultimately leading to host DNA damage (Sun et al., 2023; Zhang et al., 2023). However, whether this relationship is causal warrants further investigation. Beyond taxonomic changes, our PICRUSt2-based functional prediction provides a hypothesis-generating insight into potential mechanisms. The enrichment of metabolic pathways related to fatty acid β-oxidation, nucleotide metabolism, and energy production in the intratumoral microbiota suggests that these microbes may actively participate in and alter the local metabolic milieu. Studies have reported that *Streptococcus mutans* contributes to the progression of oral squamous cell carcinoma by reprogramming the metabolic profile of the tumor microenvironment (Zhou et al., 2024).

Several limitations of this study should be acknowledged. First, the predictive functional analysis is based on phylogenetic inference rather than direct metagenomic sequencing. Second, our study was conducted in a murine model. Although the 4NQO model recapitulates many features of human HNSCC, the translational relevance of these specific microbial findings to human patients needs to be confirmed through clinical cohort studies. Thirdly, our study used tissues from unexposed mice as normal controls, instead of matched tissues from the same animal, to model the transition from health to tumor-bearing state. Therefore, the microbial differences we observed likely combine effects of both carcinogen exposure and the tumor niche.

## 5 Conclusion

In conclusion, our integrative analysis provides compelling evidence that the intratumoral microbiota is an integral component of the tumor microenvironment in HNSCC, laying a solid foundation for further investigation into its specific role in the pathogenesis of this cancer. Modulating the intratumoral microbiota could emerge as a promising strategy to augment existing cancer therapies.

## Data Availability

The original contributions presented in the study are included in the article/supplementary material, further inquiries can be directed to the corresponding author.
